# The Efficacy and Safety of Mechanical Thrombectomy in Posterior Circulation Large Vessel Occlusion as Compared to Anterior Circulation Large Vessel Occlusion: A Systematic Review

**DOI:** 10.7759/cureus.45861

**Published:** 2023-09-24

**Authors:** Anandkumar Patel, Heet N Desai, Kofi D Seffah, Namballa Naveen, Vamsi Krishna, Safeera Khan

**Affiliations:** 1 Neurology, California Institute of Behavioral Neurosciences & Psychology, Fairfield, USA; 2 Internal Medicine, California Institute of Behavioral Neurosciences & Psychology, Fairfield, USA; 3 Internal Medicine, Piedmont Athens Regional Medical Center, Athens, USA; 4 Cardiology, California Institute of Behavioral Neurosciences & Psychology, Fairfield, USA; 5 Emergency Medicine, Steel Authority of India (SAIL) Hospital, Bokaro, IND

**Keywords:** large vessel occlusion (lvo), intracerebral hemorrhage, posterior circulation stroke, anterior circulation stroke, mechanical thrombectomy (mt)

## Abstract

Mechanical thrombectomy (MT) has been established as a standard of care for patients with stroke due to anterior circulation large vessel occlusion (AC-LVO). Due to a lack of robust evidence for the effectiveness of mechanical thrombectomy, intravenous thrombolysis (IVT) is still the only approved first-line acute reperfusion strategy for posterior circulation large vessel occlusion (PC-LVO). This systematic review analyzes and reports on the effectiveness and safety of MT in PC-LVO. A literature review was performed to identify all studies of patients with acute ischemic stroke due to PC-LVO who underwent MT with second-generation devices (stent retrievers and/or aspiration devices) that were reported between January 2017 and January 2023. The primary outcome was functional independence at 90 days, defined as a modified Rankin (mRS) score of ≤2. Secondary outcomes were successful recanalization (modified treatment in cerebral infarction score (mTICI) 2b/3), symptomatic intracerebral hemorrhage (sICH), and mortality at 90 days post-procedure. We looked at 13 studies with a total of 30,407 participants in four meta-analyses and 5951 participants in nine observational studies. In most studies, patients in the PC-LVO group were male and younger than the AC-LVO group. Higher baseline National Institutes of Health Stroke Scale (NIHSS) score, lower rates of IVT, longer onset-to-groin puncture time, lower likelihood of sICH, higher 90-day mortality rates, and higher futile recanalization rates were frequently observed in the PC-LVO group with a large discrepancy in the likelihood of functional independence at 90 days with majority studies showing comparable rates.

Hence, in patients with acute ischemic stroke caused by the PC-LVO, successful reperfusion can be achieved via MT, though at the cost of higher mortality rates. Such futile recanalization can be avoided with the refinement of procedures through technical improvements, skills training, and recognition of reliable predictors associated with it, which might help increase the efficacy of MT in PC-LVO. Additionally, future large-scale RCTs comparing patient selection and interventional strategies to avoid futile interventions are also needed.

## Introduction and background

Stroke is ranked as the second leading cause of death with an annual mortality rate of about 5.5 million, and it is the leading cause of disability worldwide [1]. It can be classified into anterior circulation stroke (ACS) and posterior circulation stroke (PCS) according to vascular territory involvement, with PCS responsible for 20% of cases [2].

Due to its varied presentation, PCS is more likely to be misdiagnosed than ACS. Depending on the blood supply and collateral status, the presentation can vary from subtle findings to devastating neurological deficits [3]. The basilar artery, which supplies most of the brain stem, occipital lobes, and part of the cerebellum and thalami, is the main artery of the posterior circulation. Patients with acute basilar artery occlusion (BAO) present with symptoms varying from isolated cranial nerve palsies or hemiplegia to locked-in syndrome, often leading to delays in reaching hospitals within golden hours of treatment [4].

The treatment strategy for acute ischemic stroke (AIS) is opening the occluded blood vessels to reestablish blood flow. The National Institute of Neurological Disorders and Stroke (NINDS) trial revolutionalized the management of AIS using recombinant tissue plasminogen activator (tPA) within three hours [5]. Eventually, the treatment window period was increased to 4.5 hours after the European Cooperative Acute Stroke Study (ECASS) III trial [6]. Currently recommended first-line therapy for AIS is still intravenous thrombolysis (IVT) using tPA when a patient reaches within 4.5 hours [7]. However, less than 10% of patients are eligible for IVT because the efficacy of IVT is time-dependent, and patients who have contraindications to tPA, received major surgery recently, or have a history of intracranial hemorrhage are ineligible [8,9]. In addition, IVT is less effective in patients with proximal large-vessel occlusion (LVO), mainly in the terminal internal carotid artery, proximal middle cerebral artery, and basilar artery, rather than in more distal occlusion [10,11]. Therefore, clinical worsening is expected in many cases of LVO unless endovascular mechanical thrombectomy (MT) is initiated.

To improve the low rates of recanalization, several new thrombolytic drugs and treatment strategies, especially mechanical thrombectomy, emerged and were proven effective [12,13]. In 2015, five randomized clinical trials (SWIFT PRIME (Solitaire™ with the Intention for Thrombectomy as Primary Endovascular Treatment for Acute Ischemic Stroke), REVASCAT (Endovascular Revascularization With Solitaire Device Versus Best Medical Therapy in Anterior Circulation Stroke Within 8 Hours), MR CLEAN (Multicenter Randomized Clinical Trial of Endovascular Treatment for Acute Ischemic Stroke in the Netherlands), EXTEND‑IA (Extending the Time for Thrombolysis in Emergency Neurological Deficits - Intra-Arterial), and ESCAPE (Endovascular Treatment for Small Core and Proximal Occlusion Ischemic Stroke)) conducted across Europe, North America, and Australia reported an overwhelming benefit of the second-generation mechanical thrombectomy devices compared to IVT in opening occluded vessels [14-18]. A subsequent meta-analysis indicated that MT was associated with significantly higher rates of angiographic revascularization and functional independence compared to standard medical care with tPA alone [12]. These findings have led to a major shift in managing patients with LVO, and MT is gradually being adopted worldwide.

However, the vessel occlusion location of patients included in the above trials was mainly in the anterior circulation, based on which American Heart Association/American Stroke Association (AHA/ASA) guidelines published in 2015 established MT as the management of choice in a patient with anterior circulation LVO (AC-LVO). No high-class evidence exists for posterior circulation LVO (PC-LVO) [19]. IVT is still the standard of care in patients with acute BAO and other large vessel occlusions in the posterior circulation [20].

Though initial studies like the Basilar Artery Occlusion Endovascular Intervention Versus Standard Medical Treatment (BEST) and the Basilar Artery International Cooperation Study (BASICS) trials both failed to demonstrate the efficacy of MT for PC-LVO, multicenter randomized control trial (RCT) of endovascular treatment for acute ischemic stroke in the Netherlands registry showed that high rates of favorable clinical outcomes and successful reperfusion could be achieved with MT for PCS despite high mortality [21-24]. The prospective multicenter Revascularization in Ischemic Stroke Patients (REVASK) registry also showed that MT in PCS had a lower risk of symptomatic intracerebral hemorrhage (sICH) and similar effectiveness compared to ACS [24]. It has also been shown that PCS patients benefit from MT beyond six hours after symptom onset and even up to 24 hours using advanced brain imaging [21]. Given the success in MT treatment of AC-LVO, neuro-interventionists routinely perform MT in PC-LVO in the absence of level-1 evidence supporting the use of thrombectomy after PCS.

Hence, this present systematic review is aimed at analyzing the available resources and evidence to determine the efficacy and safety of MT in PC-LVO as compared to AC-LVO.

## Review

Methods

This systematic review was conducted in accordance with the Preferred Reporting Items for Systematic Reviews and Meta-Analyses (PRISMA) guidelines [25].

Search Sources and Strategy

The PubMed, Cochrane Library, Science Direct, Research Gate, and Google Scholar databases were searched with no language restrictions from January 2017 to January 2023. The search strategy and keywords used for all databases were: (1) Mechanical Thrombectomy AND Posterior Circulation Stroke AND Anterior Circulation Stroke, and (2) Mechanical Thrombectomy AND Posterior Circulation Stroke AND Anterior Circulation Stroke AND Large Vessel Occlusion.

Medical Subject Headings (MeSH) search strategy was also used in PubMed as follows: ( "Thrombectomy/adverse effects"[Majr] OR "Thrombectomy/classification"[Majr] OR "Thrombectomy/education"[Majr] OR "Thrombectomy/methods"[Majr] OR "Thrombectomy/mortality"[Majr] OR "Thrombectomy/statistics and numerical data"[Majr] ) AND ( "Brain Infarction/classification"[Majr] OR "Brain Infarction/complications"[Majr] OR "Brain Infarction/diagnosis"[Majr] OR "Brain Infarction/diagnostic imaging"[Majr] OR "Brain Infarction/drug therapy"[Majr] OR "Brain Infarction/etiology"[Majr] OR "Brain Infarction/mortality"[Majr] OR "Brain Infarction/therapy"[Majr] ) AND ( "Brain Infarction/classification"[Majr] OR "Brain Infarction/complications"[Majr] OR "Brain Infarction/diagnosis"[Majr] OR "Brain Infarction/diagnostic imaging"[Majr] OR "Brain Infarction/drug therapy"[Majr] OR "Brain Infarction/etiology"[Majr] OR "Brain Infarction/mortality"[Majr] OR "Brain Infarction/physiopathology"[Majr] OR "Brain Infarction/therapy"[Majr] )

Eligibility Criteria

Studies were included based on the following criteria: (1) acute ischemic stroke patients presenting with isolated AC-LVO or PC-LVO undergoing MT with or without IVT; (2) prospective or retrospective cohort studies with patients stratified by AC-LVO and PC-LVO and a combined sample size of at least 40; (3) study must have compared baseline characteristics and reported functional outcomes using the modified Rankin Scale; (4) only English language; (5) only human studies

Studies were excluded based on the following criteria: (1) patients involved in the study received only IVT and not undergoing MT; (2) duplicated articles; (3) grey literature; (4) insufficient data for comparison purposes; (5) study with AC-LVO and PC-LVO combined into one treatment arm; (6) use of the first-generation devices (MERCI (Mechanical Embolus Removal in Cerebral Ischemia) retriever) for MT

Two researchers reviewed each study for relevance. Studies from reference lists of relevant articles were also manually searched to avoid omitting essential details. All disagreements were resolved by consensus.

Data Extraction

Two writers independently carried out the data extraction. From all qualifying studies, demographic and baseline data were gathered, including first author, publication date, participant count, age, sex, initial National Institutes of Health Stroke Scale (NIHSS), site of LVO, therapy intervention tools, onset-to-puncture time, passes, and start to reperfusion time.

Study Quality Assessment and Reducing the Risk of Bias

The Newcastle-Ottawa Scale was employed to assess the quality of the included observational studies semi-quantitatively. The maximum number of stars was nine on the Newcastle-Ottawa scale. A study with ≥ 7 stars was of high quality; otherwise, it was of low quality. Similarly, the AMSTAR checklist was used to assess the quality of the systematic review and meta-analysis. The maximum number of stars was 11 on the AMSTAR checklist. A study with ≥ 8 was of high quality; otherwise, it is of low quality. The quality of the included study was high.

Results

Study Identification and Selection

**Figure 1:**
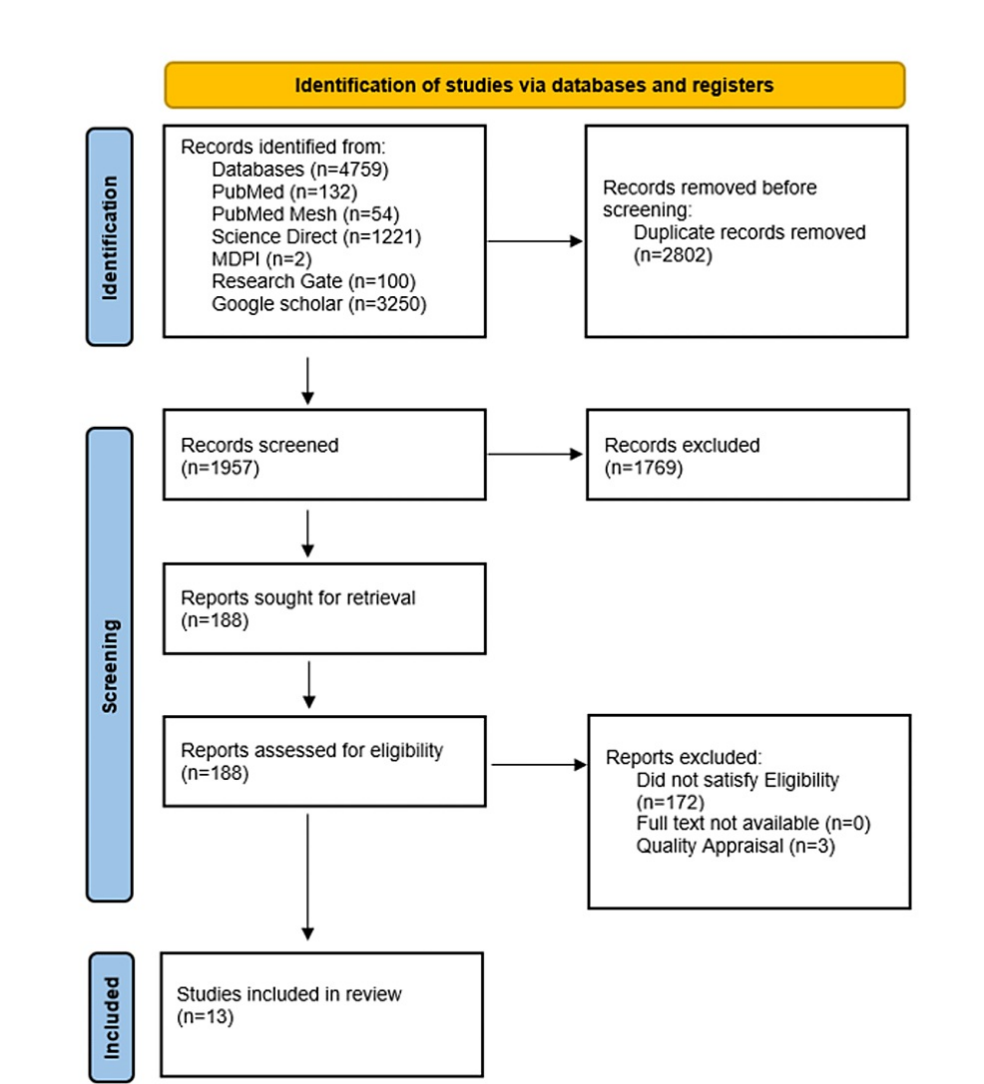
The PRISMA flowchart PRISMA: Preferred Reporting Items for Systematic Reviews and Meta-Analyses

We identified a total of 4759 relevant articles reported between 2017 and 2023 using all databases. In total, 2802 duplicate articles were removed before screening them in detail. One hundred and eighty-eight articles were shortlisted after screening these articles by going through titles and abstracts and retrieving full texts. The shortlisted full-text articles were assessed for eligibility and quality, and 13 articles were finalized for review. The selection process of the studies is shown in Figure 1 in the PRISMA flowchart.

Outcomes Measured

Primary outcome was functional independence (defined as an mRS score of ≤2) at 90 days after the procedure. Secondary outcomes were successful recanalization (mTICI 2b/3), mortality at 90 days after the procedure, and sICH.

Study Characteristics

We examined 13 studies, of which four were systematic reviews and meta-analyses, and nine were prospective/ retrospective observational studies. Out of nine observational studies, five were multicenter, and four were single-center. All of them included acute ischemic stroke patients presenting with isolated AC-LVO or PC-LVO undergoing MT with or without IVT and were assessed as separate treatment arms. In each study, a comparison of baseline characteristics like patient numbers, age, gender, baseline NIHSS score, site of LVO, initial treatment with intravenous tPA, onset to groin puncture time and outcomes in the form of successful recanalization (TICI 2b/3), sICH, functional independence at 90 days (mRS 0-2) and mortality at 90 days post-procedure was made between both arms. A total of 5951 patients were reviewed in nine observational studies where 733 patients with PC-LVO and 5218 patients with AC-LVO underwent MT. In four meta-analyses, a total of 30,407 patients were reviewed. Table 1 shows a summary and characteristics of all included studies.

**Table 1:** Summary and characteristics of all included studies ADAPT: aspiration catheter - direct aspiration first pass technique, AC: anterior circulation, ACO: anterior circulation occlusion, AIS: acute ischemic stroke, BAO: basilar artery occlusion, FR: futile recanalisation, LVO: large vessel occlusion, mRS: modified rankin scale, MT: mechanical thrombectomy, NA: not available, NIHSS: national institute of health stroke scale, PC: posterior circulation, RCT: randomised controlled trial, sICH: symptomatic intracerebral hemorrhage

Author and year of publication	Type of study	Centers	Purpose of the Study	Outcome measured	Number of participants	Devices/methods used for MT	Results	Conclusion
Hendrix et al.,2021 [26]	Retrospective observational study	Multicenter	To compare the clinical characteristics of anterior and posterior LVO strokes and identify possible predictors of successful mechanical thrombectomy outcomes for posterior LVO strokes	mRS	813	Primary aspiration or a stent retriever/aspiration catheter combination	90 days favorable functional outcomes did not differ between anterior and posterior LVO and death was significantly more associated with posterior circulation LVO thrombectomy.	Both anterior circulation and posterior circulation LVO show different clinical profiles, however mechanical thrombectomy in the posterior circulation strokes appears to have similar safety and efficacy profile in carefully selected patients.
Weber et al., 2019 [27]	Retrospective observational study	Multicenter	To assess potential procedural and outcome differences when MT with modern stent retrievers and thromboaspiration system was performed in anterior and posterior circulation large vessel occlusions	mRs	1100	Stent retriever and aspiration catheter	In the mechanical thrombectomy procedure, favorable functional outcomes at three months and mortality did not significantly differ between large vessel anterior and posterior circulation strokes.	The use of MT is supported in acute ischemic stroke patients with occlusion of the basilar artery, as well as in patients with significant clinical deficits due to occlusion of the vertebral and posterior cerebral arteries.
Alaweih et al., 2017 [28]	Retrospective observational study	single-center	To analyze the variations in neurological outcomes, mortality, hospital stay, and complication rates between posterior and anterior circulation thrombectomy	mRs	436	Aspiration catheter - direct aspiration first pass technique (ADAPT) ± stent retriever	Posterior circulation thrombectomy did not show pre and post-procedural differences compared to thrombectomy in anterior circulation stroke.	Patients presenting with posterior LVOs and AIS benefit equally from thrombectomy with ADAPT compared with those with anterior circulation LVOs.
Huo et al., 2020 [29]	Retrospective observational study	Multicenter	Examine the distinctions between anterior circulation stroke and posterior circulation stroke in terms of their traits and determine how well they will respond to thrombectomy	mRS	741	Stent retriever, aspiration, Balloon angioplasty, Permanent stenting, Intra-arterial thrombolysis	Three-month follow-up outcomes showed that anterior circulation stroke patients had better functional independence and lower mortality. However, symptomatic intracerebral hemorrhage was more common in anterior circulation stroke.	The endovascular treatment effectively reduces mortality and improves functional outcomes in patients with acute large vessel occlusion. It also indicates that the severity of symptoms and the presence of comorbidities play an important role in determining the treatment outcome.
De Lecinana et al., 2016 [30]	Retrospective Observational study	Multicenter	To analyze the efficacy and safety of mechanical thrombectomy for basilar artery thrombosis, and compare the outcomes with those of anterior circulation occlusions	mRS	479	NA	Patients with basilar artery occlusion had a high rate of procedural complications. At three months, patients with BAO had a lower rate of independence and high mortality.	Performing mechanical thrombectomy on PC strokes is more time-consuming and laborious than AC strokes. Older age and longer procedure duration make it more likely that the procedure will be unsuccessful in BAO cases.
Meinel et al., 2019 [31]	Retrospective observational study	Multicenter	To compare patients' outcomes, relative merits of achieving recanalization, and predictors of futile recanalization (FR) between BAO and anterior circulation large vessel occlusion (AC-LVO) MT	mRS	1739	Stent retriever	MT in BAO had a similar success rate in terms of mortality and sICH as AC-LVO, with no considerable difference in overall favorable outcome when accounting for any baseline variations.	In selected patients, similar outcomes can be achieved in BAO and AC-LVO patients treated with MT. Randomized controlled trials comparing patient selection and interventional strategies seem warranted to avoid FR.
Hu et al., 2017 [32]	Retrospective observational study	single-center	To analyze the outcomes of stent retriever mechanical thrombectomy for BAO, compared with anterior circulation occlusions (ACO)	mRS	161	Stent retriever	No major variations existed among the two sets of individuals regarding difficulties. However, the BAO group had a higher mortality rate as compared to the ACO group.	MT of BAO patients showed worse clinical outcomes and higher mortality rates than ACO patients. However, MT with a stent retriever in BAO is an effective treatment because successfully recanalized patients showed good clinical outcomes in BAO patients.
Singh et al., 2017 [33]	Retrospective observational study	Multicenter	To analyze the efficacy of mechanical thrombectomy as a treatment for acute ischemic stroke in 137 patients	mRS	137	Stent retriever	No difference in efficacy and outcome of MT in AC and PC stroke. Despite delayed presentation and high NIHSS score in PC stroke, three months outcome was comparable with AC stroke.	Solitaire FR is safe and achieves good revascularization rates and functional outcomes in patients with AIS and large artery occlusion. MT is feasible in a real‑world setting in India, and results are comparable to RCT.
Uno et al., 2019 [34]	Prospective observational study	single-center	To investigate the clinical effectiveness of MT for BAO and to ascertain whether outcomes for patients with BAO were comparable to those with anterior circulation large vessel occlusion (AC-LVO)	mRS	345	Stent retriever, aspiration or both	No significant difference was noted in favorable outcomes (modified Rankin Scale score ≤2) between BAO and AC-LVO patients.	MT in BAO and AC-LVO patients resulted in a high reperfusion rate with favorable outcomes in approximately half of the patients. MT may be considered the standard care for BAO patients.
Wang et al., 2020 [35]	Meta-analysis	NA	To examine MT results in PC-LVO and compare these results to AC-LVO to provide basic information for subsequent research.	mRS	3979	NA	In comparison to AC-LVO, thrombectomy in PC-LVO showed a lower rate of sICH and functional independence and a higher rate of mortality.	The result for MT was poor in PC-LVO. There were lower rates of sICH and high recanalization rates, given that MT may serve as an adjunct to standard treatment.
Zhao et al., 2020 [36]	Systematic review and meta-analysis	NA	Aimed to compare the safety and effectiveness of MT in PC-LVO to AC-LVO by methodically analyzing the evidence already available	mRS	4619	NA	In a preliminary analysis, MT in patients with PC-LVO compared to patients with AC-LVO had a lower incidence of sICH, higher mortality, and worse functional outcome.	Patients with pc-LVO who get MT had a lower risk of sICH but appeared to have a worse prognosis. However, this study has several limitations, and additional randomized trials must be conducted to assess this question properly.
Adusumilli et al. 2022 [37]	Systematic review and meta-analysis	NA	To compare the technical success and functional outcomes of MT in PC-LVO versus AC-LVO patients	mRS	12911	NA	AC-LVO and PC-LVO patients had comparable rates of successful recanalization. However, the AC-LVO group had greater odds of 90-day functional independence and lower odds of 90-day mortality.	MT achieves similar recanalization rates with a similar safety profile in PC-LVO and AC-LVO patients. Patients with PC-LVO are less likely to achieve functional independence after MT. Future studies should identify PC-LVO patients who are likely to achieve favorable functional outcomes.
Mbroh et al. 2021 [38]	Systematic review and meta-analysis	NA	To study the extent to which there was a difference in MT safety and efficacy outcomes of PC-LVO from AC-LVO	mRS	8898	NA	There was no significant difference between AC-LVO and PC-LVO regarding successful canalization and favorable functional outcome at 90 days. However, PC-LVO was associated with lower sICH and higher mortality.	Similar to AC-LVO, MT in PC-LVO may be equally effective in achieving successful recanalization and a positive functional result at 90 days.

Discussion

Strokes occurring due to PC-LVO and AC-LVO differ in terms of their pathophysiology, clinical features, and the outcome of their acute management with endovascular MT. Limited sample size and few RCTs including PC-LVO patients make the safety and efficacy of MT difficult to assess in the PC-LVO group, which has been confirmed in AC-LVO.

The morbidity and mortality rates of conservatively managed acute BAO (largest subgroup in PC-LVO) can reach up to 80-90% [39], which is the highest among all types of ischemic stroke. Patients who enroll in RCTs are seeking treatment and hence, they should not be randomized to a conservative treatment group which is logically known to be inferior to MT, given its success in AC-LVO. In AC-LVO, MT has been accepted in most clinical settings as the best way to obtain recanalization. Therefore, randomizing patients with PC-LVO into groups including no-MT is considered mostly unethical.

Among the few RCTs that focused on PCS, the BEST RCT was terminated due to loss of equipoise, resulting from a high crossover rate and was topped by a small sample size. This trial reported no difference in favorable outcomes between MT patients and those receiving only standard medical treatment, including IVT [22]. Similarly, another RCT, the BASICS trial, failed to demonstrate MT's efficacy for PC-LVO.

Recently, a few studies have shown that MT in the posterior circulation appears to have a similar safety and efficacy profile in carefully selected patients. Still, such studies are underpowered and provide lower-quality evidence to guide PC-LVO treatment decisions [23,24].

Therefore, we conducted a systemic review of studies done over five years (2017 to 2023) to study the extent to which MT in PC-LVO differs from that in AC-LVO. Patients in these studies underwent MT using second-generation MT devices which were introduced in the last decade with proven superiority to first-generation MT devices.

Baseline Characteristics of PC-LVO versus AC-LVO

Results of characteristics like age, gender, baseline NIHSS, and site of LVO for the included studies are reported in Table 2 and Table 3.

**Table 2:** Demographics and baseline characteristics - Observational studies ACA: anterior cerebral artery, AC-LVO: anterior circulation large vessel occlusion, BA: basilar artery, ICA: internal carotid artery, NA: not available, NIHSS: national institute of health stroke scale, PCA: posterior cerebral artery, PC-LVO: posterior circulation large vessel occlusion, VA: vertebral artery

Studies	Sample Size			Age (in years)		Male (%)		Baseline NIHSS		Occluded vessel	
	Total	PC-LVO	AC-LVO	PC-LVO	AC-LVO	PC-LVO	AC-LVO	PC-LVO	AC-LVO	PC-LVO	AC-LVO
Hendrix et al. [26]	813	77 (9.4%)	736 (90.5%)	72 (59-81)	75 (63-83)	39/77 (50.6%)	334/736 (45.4%)	22 (14-29)	19 (13-23)	VA -8 (10.4%), BA -59 (76.6%), PCA- 10 (13%)	ICA -190 (25.8%), M1- 436 (59.2%), M2- 93 (12.6%), M3- 10 (1.4%), ACA- 7(1%)
Weber et al. [27]	1100	139 (12.6%)	961 (87.3%)	65.4 ± 15.8	69.0 ± 13.9	82/139 (59%)	498/961 (51.9%)	12 (6-21)	15 (12-19)	BA-118 (84.9%), VA-15 (16.5%), PCA-6 (4.3%)	NA
Alaweih et al. [28]	436	56 (12.8%)	380 (87.1%)	27 ± 48	67 ± 15	48/56 (85.7%)	188/380 (49.4%)	17.4 ± 11	15.3 ± 7	NA	NA
Huo et al. [29]	741	145 (19.5%)	596 (80.5%)	64.3 ± 12.8	63.7 ± 14.0	108/145 (74.5%)	380/596 (63.8%)	20 (11–26)	16 (12–21)	BA- 80, VA- 58, PCA- 7	NA
De Lecinana et al. [30]	479	52 (10.8%)	427 (89.2%)	64 (50-74)	70 (60-77)	35/52 (67%)	213/427 (50%)	11 (6-23)	18 (14-21)	BA-52	ICA -100 (21%), M1-244 (51%), M2-40 (8%), tandem occlusion -43 (9%)
Meinel et al. [31]	1739	165 (9.4%)	1574 (90.6%)	70 (59-80)	73 (61-82)	96/165 (58.2%)	764/1574 (48.5%)	18 (8-30)	17 (12-20)	BA - 165	NA
Hu et al. [32]	161	24 (14.9%)	137 (85.1%)	65.7 (32-85)	65.5 (22-87)	13/24 (54.2%)	78/137 (56.9%)	14.2 (2-34)	10.4 (3-26)	BA - 24	MCA-94 (68.7%), ICA-42 (30.6%), ACA-1 (0.7%)
Singh et al. [33]	137	25 (18.2%)	112 (81.8%)	56.4 ± 9.19	57.85 ± 12.52	16/25 (64%)	71/112 (63.4%)	19 ± 5.5	15.5 ± 4.32	BA - 25	M1-61 (54.4%), ICA-51 (45.5%)
Uno et al. [34]	345	50 (14.4%)	295 (85.6%)	72.5 (64.75-78.5)	77 (69-84)	33/50 (66%)	144/295 (48.8%)	24.5 (13 – 32)	18 (13 – 22)	BA - 50	NA

**Table 3:** Demographics and baseline characteristics - Observational studies (continued) AC-LVO: anterior circulation large vessel occlusion, IV: intravenous, MT: mechanical thrombectomy, NA: not available, PC-LVO: posterior circulation large vessel occlusion, t-PA: tissue plasminogen inhibitor

Studies	IV tPA (%)		Last known well time to groin puncture (min)		Procedural time (min)		Number of thrombectomy maneuvers		Time from onset to recanalization/end of MT (min)	
	PC-LVO	AC-LVO	PC-LVO	AC-LVO	PC-LVO	AC-LVO	PC-LVO	AC-LVO	PC-LVO	AC-LVO
Hendrix et al. [26]	28/77 (36.4%)	349/736 (47.4%)	269 (150-683)	229 (160-365)	NA	NA	NA	NA	NA	NA
Weber et al. [27]	58/139 (41.7%)	505/961 (52.5%)	225 (160-425)	195 (140-282)	NA	NA	2 (1-3)	2 (1-3)	329 ± 259	274 ± 190
Alaweih et al. [28]	19/56 (33.9%)	153/380 (40.3%)	480.3 ± 845.6	491.7 ± 812.5	NA	NA	3.1 (2.4)	2.9 (2.1)	NA	NA
Huo et al. [29]	25/145 (17.2%)	187/596 (31.5%)	339 (225–431)	260 (190–350)	NA	NA	NA	NA	434 (300–530)	360 (290–440)
De Lecinana et al. [30]	20/52 (38.5%)	220/427 (51.5%)	312 (225–510)	280 (212–365)	100 (40–130)	60 (39–90)	2 (1-4)	2 (1-3)	385 (320–540)	315 (240–415),
Meinel et al. [31]	71/165 (43.0%)	779/1574 (49.5%)	300 (211–480)	225 (165–315)	45 (30–81)	47 (30–75)	1 (1-2)	2 (1-3)	NA	NA
Hu et al. [32]	9/24 (34.0%)	64/137 (46.7%)	268 (59–552)	216 (36–612)	122 (40–285)	116 (27–360)	2.0 (1-6)	2.7 (1-10)	390 (137–675)	333 (90–870)
Singh et al. [33]	7/25 (28.0%)	62/112 (55.4%)	326.4 ± 191.8	220 ± 80.6	NA	NA	1.6±0.65	1.54±0.78	NA	NA
Uno et al. [34]	26/50 (52.0%)	170/295 (57.6%)	NA	NA	27.5 (12.75-66.5)	39 (19.5-61)	NA	NA	200 (160-262)	220 (174-297.5)

Age and gender: In most trials, patients in the PC-LVO group were younger than AC-LVO, which was statistically significant, as shown in studies by Weber et al., De Lecinana et al., Meinel et al., and Uno et al. [27,30,31,34]. Also, in the meta-analysis by Wang et al. and Mbroh et al., patients in the PC-LVO cohort were younger [35,38].

Further results showed that males dominated the PC-LVO group, as observed in all nine observational studies except Hu et al. [32]. This finding was also statistically significant in studies by Weber et al., Huo et al., De Lecinana et al., Meinel et al., and Uno et al. [27,30,31,34].

Site of occlusion: In the studies where occlusion sites were reported, the middle cerebral artery (M1 division) was the most prevalent site of AC-LVO. BAO was the predominant lesion location in the PC-LVO. Consecutive LVO case series have reported PC-LVOs to account for <10% of all LVOs [19,40]; however, in our analysis, larger thrombectomy cohorts studied by Weber et al., Alaweih et al., Huo et al., De Lecinana et al., Hu et al., Singh et al., and Uno et al. amounted to approximately 10-20% PC-LVO thrombectomies [27,29,30,32-34]. Although these data are highly biased by patient selection and study design, they indicate that PC-LVO thrombectomy represents a standard neuro-interventional procedure despite its current lack of high-class recommendations and level of evidence.

Baseline NIHSS: Baseline pre-thrombectomy NIHSS was higher in patients who underwent MT for the PC-LVO compared with AC-LVO in studies by Hendrix et al., Alaweih et al., Huo et al., Meinel et al., Hu et al., Singh et al., Uno et al. as well as in the meta-analysis by Mbroh et al. [26,28,29,31-34,38]. In contrast, a higher baseline NIHSS in AC-LVO than in PC-LVO has been reported by Weber et al. and De Lecinana et al. [27,30]. In the meta-analysis by Wang et al., baseline pre-thrombectomy NIHSS varied [35]. The reason for this discrepancy could be partly explained by the NIHSS being highly weighted toward deficits occurring in AC-LVO, such as aphasia and hemiparesis, and neglecting PC-LVO symptomatology, such as unsteady gait, dysphagia, and oculomotor deficits [24]. On the other hand, once posterior circulation occlusion (PCO) leads to a disturbance of consciousness, the NIHSS score will increase significantly. Therefore, for a given NIHSS score, patients with PC-LVO may have a more serious condition than patients with AC-LVO.

IVT rates: PC-LVO patients received IVT less often than AC-LVO patients, as shown in all the included nine observational studies and meta-analyses by Wang et al. and Mbroh et al. [35,38]. The most reasonable explanation for this is IVT being a time-dependent treatment and the varied symptomatology of PC-LVO strokes. Prodromal symptoms are seen in up to 60% of cases of PC-LVO, which is a reason for misdiagnosis and wrong specialty consultation in many cases [4,40]. Visual deficits, ataxia, giddiness, and decreased level of consciousness, the most common signs and symptoms of acute PC-LVO, can confuse clinicians with "stroke chameleons" [41-43]. As a result, PC-LVO patients may not succeed in presenting within the widely accepted 4.5-hour time window to receive IVT [44]. Also, patients with PCS are often in severe condition when admitted to the hospital, often missing the opportunity for acute treatment [45].

Onset-to-groin puncture times: Delay in MT in patients with PC-LVO was reflected in the longer onset-to-groin puncture times, which was observed in all the included observational studies. It varied between 225 minutes (Weber et al.) to 480.3 minutes (Alaweih et al.) [27,28]. It was also statistically significant in studies by Weber et al., Huo et al., Meinel et al., and Hu et al. [27,29,31,32]. A meta-analysis by Mbroh et al. also showed significantly longer onset-to-groin puncture time in the PC-LVO group (standardized mean difference (SMD) = 0.59, 95%CI 0.33-0.85, p < 0.00001) [38]. The same argument for why PC-LVO patients present as stroke chameleons and are misdiagnosed, delaying definitive care, can be used to explain this observation.

PC-LVO versus AC-LVO Thrombectomy: Technical Efficacy

Successful recanalization (mTICI 2b/3 grade recanalization): As shown in Table 4, there was no statistically significant difference in rates of successful recanalization (defined as mTICI 2b/3 grade recanalization) between PC-LVO and AC-LVO cohorts in all the observational studies included in this analysis except Meinel et al., where they showed higher odds of successful recanalization in PC-LVO cohort (OR=2.740, 95%CI 1.145-6.554, p=0.011) [31]. This exceptionally high rate was explained by advances in MT technique, operator adjudication, use of thrombolysis in cerebral infarction (TICI) score in PC-LVO in their registry, and different patient selection methods [31]. Also, successful recanalization rates were comparable in all four meta-analyses included in the review as shown in Table 5.

**Table 4:** Safety and Outcomes - Observational studies AC-LVO: anterior circulation large vessel occlusion, mRS: modified rankin scale, mTICi: modified treatment in cerebral infarction, PC-LVO: posterior circulation large vessel occlusion, sICH: sypmtomatic intracranial hemorrhage

Safety and Outcomes								
Studies	sICH		Successful Recanalization (mTICI 2b/3)		mRS 0–2 at 90 days		Mortality at 90 days	
	PC-LVO	AC-LVO	PC-LVO	AC-LVO	PC-LVO	AC-LVO	PC-LVO	AC-LVO
Hendrix et al. [26]	NA	NA	63/77 (81.8%)	639/736 (86.8%)	32/77 (41.6%) (mRS 0-3)	380/736 (51.6%) (mRS 0-3)	31/77 (40.3%)	198/736 (26.9%)
Weber et al. [27]	0	29/961 (3%)	96/139 (71.6%)	719/961 (76.0%)	35/139 (38.0%)	281/961 (42.6%)	31/139 (33.7%)	203/961 (30.8%)
Alaweih et al. [28]	NA	NA	54/56 (96.4%)	351/380 (92.4%)	24/56 (42.9%)	164/380 (43.2%)	16/56 (28.6%)	68/380 (17.9%)
Huo et al. [29]	4/145 (2.8%)	44/596 (7.4%)	119/145 (82.1%)	520/596 (87.3%)	44/145 (30.3%)	279/596 (46.8%)	49/145 (33.8%)	98/596 (16.4%)
De Lecinana et al. [30]	1/52 (2%)	23/427 (5%)	39/52 (75%)	359/427 (84%)	21/52 (40%)	237/427 (58%)	17/52 (33%)	48/427 (12%)
Meinel et al. [31]	8/165 (4.8%)	98/1574 (6.3%)	149/165 (90.3%)	1299/1574 (82.7%)	55/165 (33.3%)	604/1574 (38.3%)	55/165 (36.2%)	344/1574 (24.4%)
Hu et al. [32]	1/24 (4.1%)	12/137 (8.7%)	19/24 (79.1%)	110/137 (80.2%)	NA	NA	4/24 (16.6%)	8/137 (5.8%)
Singh et al. [33]	NA	NA	21/25 (84.0%)	104/112 (92.9%)	12/25 (48.0%)	71/112 (63.4%)	2/25 (8.0%)	7/112 (6.3%)
Uno et al. [34 ]	0 (0%)	9 (3.1%)	50/50 (100%)	250/295 (84.7%)	27/42 (64.3%)	105/213 (49.3%)	4/42 (9.5%)	22/213 (10.3%)

**Table 5:** Safety and Outcomes – meta-analyses AC-LVO: anterior circulation large vessel occlusion, CI: confidence interval, mTICi: modified treatment in cerebral infarction, OR: odds ratio,  PC-LVO: posterior circulation large vessel occlusion

Outcomes of PC-LVO versus AC-LVO thrombectomy												
Studies	Sample size		Symptomatic intracranial hemorrhage		Successful recanalization (mTICI 2b/3)		Functional independence at 90 days		Mortality		Futile recanalization	
	PC-LVO	AC-LVO	PC-LVO	AC-LVO	PC-LVO	AC-LVO	PC-LVO	AC-LVO	PC-LVO	AC-LVO	PC-LVO	AC-LVO
Wang et al. [35]	474	3505	10/448 (2.23%)	191/3457 (5.53%)	382/474 (80.6%)	2821/3502 (80.6%)	123/368 (33.4%)	1193/2909 (41.0%)	119/448 (26.6%)	634/3469 (18.3%)	91/191 (47.6%)	561/1642 (34.2%)
			OR=0.54 (95% CI, 0.29–0.99, p=0.05)		OR =1.01 (95% CI, 0.62–1.65, p=0.95)		OR =0.72 (95% CI, 0.57–0.90, p=0.005)		OR= 2.03 (95% CI,1.30–3.18, p=0.002)		OR=1.75 (95% CI,1.30 2.37, p=0.003)	
Zhao et al. [36]	563	4056	10/423 (2.3%)	180/3357 (5.3%)	455/554 (82.1%)	3252/4000 (81.3%)	135/412 (32.7%)	1286/3342 (38.4%)	135/504 (26.7%)	702/3859 (18.1%)	NA	NA
			OR= 0.48 (95% CI, 0.26-0.88, p = 0.02)		OR=1.12 (95%CI, 0.88- 1.421 ,p = 0.35)		OR=1.12 (95%CI, 0.88- 1.421 ,p = 0.35)		OR=1.8 (95% CI, 1.37-2.87, p= 0.0003)			
Mbroh et al. [37]	1172	7726	17/674 (2.5%)	264/4611 (5.7%)	884/1067 (82.8%)	5864/7130 (82.2%)	276/685 (40.2%)	2134/5122 (41.6%)	270/896 (30.13%)	1392/6189 (22.49%)	NA	NA
			OR = 0.44 (95% CI, 0.27–0.71, p = 0.0008)		OR= 1.03 (95% CI, 0.76–1.41, p = 0.83)		OR = 0.97 (95% CI 0.73–1.27, p = 0.80)		OR = 1.82 (95% CI, 1.33–2.48, p = 0.0002)			
Adusumilli et al.* [38]	1612	11299	21/723 (2.9%)	271/4884 (5.54%)	1309/1593 (82.1%)	9246/11189 (82.6%)	580/1355 (42.8%)	4337/8984 (48.2%)	348/1129 (30.8%)	1794/7755 (23.1%)	NA	NA
			OR = 0.11( 95% CI, 0.01- 0.201, p=0.030)		OR =0.92 [95% CI,0.65–1.32, p=0.644)		OR =1.26 (95% CI ,1.00; 1.59, p= 0.05)		OR =0.58 [95% CI,0.43- 0.79, p=0.002)			

Time from onset to recanalization (OTR): There was a significant difference in OTR between both arms ranging 329 to 434 minutes for PC-LVO and 274 to 360 minutes for AC-LVO, as reflected in studies done by Weber et al., Huo et al., De Lecinana et al., and Hu et al.. An exception was the observational study by Uno et al. where OTR was 200 minutes (160-262) for PC-LVO and 220 minutes (174-297.5) for AC-LVO [27,29,30,32,34]. This difference reflects the long delay from onset-to-groin puncture times and the higher complexity of the procedures, including the need for stenting in those with BAO. The meta-analysis by Wang et al. and Mbroh et al. also showed similar findings [35,38].

Procedural time: MT in PC-LVO took a long time for completion compared to AC-LVO, as shown by De Lecinana et al. (100 minutes (40-130) vs. 60 minutes (39-90), p=0.006), whereas there wasn't any significant difference according to data presented by Meinel et al. (45 minutes (30-81) vs. 47 minutes (30-75), p= 0.824) and Hu et al. (122 minutes (40-285) vs. 116 minutes (27-360), p=0.271) [30-32].

PCO versus ACO Thrombectomy: Safety

sICH: The likelihood of sICH, which was defined as any intracranial hemorrhage associated with an increase of 4 points or more on the NIHSS score, was significantly lower in PC-LVO compared to AC-LVO as shown in all the four included meta-analyses. A similar finding was observed in all the included observational studies, although not in a statistically significant range.

We can try to explain why patients with PC-LVO receiving MT have a lower rate of sICH than patients with AC-LVO with the following points: (a) compared to anterior circulation, posterior circulation vessels are mostly small end-arteries, with relatively less high perfusion and less reperfusion injury than anterior circulation vessels [46,47], (b) there is more hemodynamic flow in the carotid artery than the vertebral artery; thus, the vascular bed pressure is higher in the anterior circulation system, (c) the infarction area occurred in PCS is usually relatively small than that in ACS, which may decrease the risk of bleeding events [48,49], (d) abundance of collateral circulation existing in posterior circulation leads to slower evolution of irreversible ischemia and, 5) lower IVT rate in PC-LVO group leading to fewer rates of sICH.

PC-LVO versus AC-LVO Thrombectomy: Outcome

Favorable functional outcome (mRS 0-2) at 90 days: There was a large discrepancy in the likelihood of favorable functional outcomes seen after MT in PC-LVO compared to AC-LVO, as shown in Table 4 and Table 5. A meta-analysis by Mbroh et al. and all the included observational studies except Huo et al. and De Lecinana et al. showed comparable rates of functional independence at 90 days, which means that a favorable functional outcome in PC-LVO is equally possible just as in AC-LVO despite the longer onset-to-IVT and onset-to-groin puncture times. Penumbra in posterior circulation stroke persists for a longer time compared to anterior circulation stroke, possibly due to better collateralization in the brainstem, which was the hypothesis put forward to explain this finding [48]. Hence, shorter onset-to-IVT and onset-to-groin puncture times could influence a better functional MT outcome in PC-LVO. Huo et al. and De Lecinana et al. showed worse 90-day functional outcomes after MT for PC-LVO, which could be due to higher baseline severity of stroke at admission, laborious and time-consuming endovascular procedures, a tendency towards a higher rate of complications related with the procedures and higher mortality rates in studied patients [29,30].

Mortality (mRS 6) at 90 days: MT in PC-LVO was associated with a significantly higher mortality likelihood than AC-LVO, as shown in all four meta-analyses. Similarly, 90-day mortality rates were also significantly higher in the PC-LVO group in all the observational studies except Weber et al., Alaweih et al., and Uno et al., where the rates were comparable [27,28,34]. This higher 90-day mortality in PC-LVO patients can result from higher baseline NIHSS, which is suggestive of higher stroke severity on admission. It has been consistently shown as an important predictor of bad stroke outcomes, especially in the posterior circulation [50,51]. Here, many studies only included patients with BAO for the PC-LVO group, and in the rest of the others, the basilar artery was the most frequent site of PC-LVO. Stroke due to BAO has been described as the most severe in relation to other occlusion sites in PC-LVO, which may explain this higher mortality [52]. In addition, the higher mortality rate in PC-LVO might be attributable to the damage occurring to the vital brainstem centers leading to a deep comatose state, dysphagia, respiration difficulties leading to ventilatory support, tracheostomy, pneumonia, and complications due to long-term bedridden state. Although it is agreed upon that younger patients tend to have a better stroke outcome than older patients, this study demonstrates that although PC-LVO patients are younger, they have a greater mortality rate than AC-LVO patients.

Futile recanalization: Futile recanalization, defined as the poor functional outcome with mRS 4-6, despite successful recanalization by MT, has been reported in individual studies as being significantly higher in PC-LVO than AC-LVO [31,35]. In the study by Meinel et al., futile recanalization was found more often in BAO, with 47% and 34% of patients achieving futile recanalization in PC-LVO and AC-LVO groups, respectively (adjusted OR (aOR)=2.146, 95%CI 1.267-3.633, p=0.002) [31]. Similar results were found in a meta-analysis by Wang et al., who found that the PC-LVO group had a 1.75 times higher risk of futile recanalization (OR-1.75; 95%CI 1.30-2.37, p=0.003) [35].

With such a higher rate of futile recanalization associated with MT in PC-LVO, ethics and patient preferences should be considered when considering the risk of subjecting patients to a treatment that may avoid death but instead creates long-term dependence. Recognition of factors associated with futile recanalization in PC-LVO might help increase the efficacy of MT in such patients. Efforts have been made to identify predictors of futile recanalization in PC-LVO and it has been shown that age, stroke severity on admission, duration of the procedure, and intracranial stenting are significant predictors [31,30].

Thus, the most important question is not whether to treat PC-LVO patients with MT or not, but rather which patients' recanalization would be futile and cause long-term dependence. Consequently, conducting RCTs that compare patient selection strategies with advanced imaging methods and concurrent medical therapy is vitally necessary.

Limitations

In our systemic review, most of the studies included were retrospective and observational, which does not allow for the generalization of our results. Also, there was a huge disparity in the number of PC-LVO and AC-LVO patients. The fact that thrombectomy is less commonly performed in PCS than in ACS patients resulted in fewer patients in the PC-LVO group. This leads to inadequate statistical power to assess the impact of endovascular treatment on major clinical outcomes between the two groups, especially in mortality.

Apart from that, non-transparent patient selection with disparities in patient inclusion criterion and definition of outcomes, different baseline characteristics, and heterogeneous endovascular treatments are the potential sources of bias. Apart from the type of devices used, the prognosis of patients could also be affected by the different perioperative management and procedures of MT. Therefore, follow-up multicenter randomized controlled studies are needed to validate the findings presented in this work.

## Conclusions

Outcomes of MT in PC-LVO are far from ideal, with higher 90-day mortality rates and higher futile recanalization rates, although the rates of sICH appear to be lower compared to AC-LVO. On the other hand, the morbidity and mortality rates of conservatively managed acute BAO can reach up to 80-90%, which is the highest among all types of ischemic stroke. Hence, MT in PC-LVO can become a gold standard procedure if refinements can be made through technical improvements and skills training, as well as recognition of reliable predictors associated with futile recanalization, leading to increased overall efficacy. To achieve these goals, large-scale RCTs comparing patient selection and interventional strategies to avoid futile interventions are needed in the future.

## References

[1] Lopez AD, Mathers CD, Ezzati M, Jamison DT, Murray CJ (2006). Global and regional burden of disease and risk factors, 2001: systematic analysis of population health data. Lancet.

[2] Sacks D, Baxter B, Campbell BC (2018). Multisociety consensus quality improvement revised consensus statement for endovascular therapy of acute ischemic stroke. Int J Stroke.

[3] Searls DE, Pazdera L, Korbel E, Vysata O, Caplan LR (2012). Symptoms and signs of posterior circulation ischemia in the new England medical center posterior circulation registry. Arch Neurol.

[4] van der Hoeven EJ, Schonewille WJ, Vos JA (2013). The Basilar Artery International Cooperation Study (BASICS): study protocol for a randomised controlled trial. Trials.

[5] Robinson T, Zaheer Z, Mistri AK (2011). Thrombolysis in acute ischaemic stroke: an update. Ther Adv Chronic Dis.

[6] Kurre W, Bansemir K, Aguilar Pérez M, Martinez Moreno R, Schmid E, Bäzner H, Henkes H (2016). Endovascular treatment of acute internal carotid artery dissections: technical considerations, clinical and angiographic outcome. Neuroradiology.

[7] Powers WJ, Rabinstein AA, Ackerson T (2018). 2018 guidelines for the early management of patients with acute ischemic stroke: a guideline for healthcare professionals from the American Heart Association/American Stroke Association. Stroke.

[8] Lees KR, Bluhmki E, von Kummer R (2010). Time to treatment with intravenous alteplase and outcome in stroke: an updated pooled analysis of ECASS, ATLANTIS, NINDS, and EPITHET trials. Lancet.

[9] Emberson J, Lees KR, Lyden P (2014). Effect of treatment delay, age, and stroke severity on the effects of intravenous thrombolysis with alteplase for acute ischaemic stroke: a meta-analysis of individual patient data from randomised trials. Lancet.

[10] Lee KY, Han SW, Kim SH (2007). Early recanalization after intravenous administration of recombinant tissue plasminogen activator as assessed by pre- and post-thrombolytic angiography in acute ischemic stroke patients. Stroke.

[11] Bhatia R, Hill MD, Shobha N (2010). Low rates of acute recanalization with intravenous recombinant tissue plasminogen activator in ischemic stroke: real-world experience and a call for action. Stroke.

[12] Badhiwala JH, Nassiri F, Alhazzani W (2015). Endovascular thrombectomy for acute ischemic stroke: a meta-analysis. JAMA.

[13] Huang X, Cheripelli BK, Lloyd SM (2015). Alteplase versus tenecteplase for thrombolysis after ischaemic stroke (ATTEST): a phase 2, randomised, open-label, blinded endpoint study. Lancet Neurol.

[14] Saver JL, Goyal M, Bonafe A (2015). Stent-retriever thrombectomy after intravenous t-PA vs. t-PA alone in stroke. N Engl J Med.

[15] Jovin TG, Chamorro A, Cobo E (2015). Thrombectomy within 8 hours after symptom onset in ischemic stroke. N Engl J Med.

[16] Goyal M, Demchuk AM, Menon BK (2015). Randomized assessment of rapid endovascular treatment of ischemic stroke. N Engl J Med.

[17] Campbell BC, Mitchell PJ, Kleinig TJ (2015). Endovascular therapy for ischemic stroke with perfusion-imaging selection. N Engl J Med.

[18] Berkhemer OA, Fransen PS, Beumer D (2015). A randomized trial of intraarterial treatment for acute ischemic stroke. N Engl J Med.

[19] Alexandre AM, Valente I, Consoli A (2021). Posterior circulation endovascular thrombectomy for large vessels occlusion in patients presenting with NIHSShss score ≤ 10. Life (Basel).

[20] Schonewille WJ, Wijman CA, Michel P (2009). Treatment and outcomes of acute basilar artery occlusion in the Basilar Artery International Cooperation Study (BASICS): a prospective registry study. Lancet Neurol.

[21] Langezaal LC, van der Hoeven EJ, Mont'Alverne FJ (2021). Endovascular therapy for stroke due to basilar-artery occlusion. N Engl J Med.

[22] Liu X, Dai Q, Ye R (2020). Endovascular treatment versus standard medical treatment for vertebrobasilar artery occlusion (BEST): an open-label, randomised controlled trial. Lancet Neurol.

[23] Pirson FA, Boodt N, Brouwer J (2022). Endovascular treatment for posterior circulation stroke in routine clinical practice: results of the multicenter randomized clinical trial of endovascular treatment for acute ischemic stroke in the Netherlands Registry. Stroke.

[24] Yu W, Higashida RT (2022). Endovascular thrombectomy for acute basilar artery occlusion: latest findings and critical thinking on future study design. Transl Stroke Res.

[25] Page MJ, McKenzie JE, Bossuyt PM (2021). The PRISMA 2020 statement: an updated guideline for reporting systematic reviews. BMJ.

[26] Hendrix P, Killer-Oberpfalzer M, Broussalis E (2022). Outcome following mechanical thrombectomy for anterior circulation large vessel occlusion stroke in the elderly. Clin Neuroradiol.

[27] Weber R, Minnerup J, Nordmeyer H, Eyding J, Krogias C, Hadisurya J, Berger K (2019). Thrombectomy in posterior circulation stroke: differences in procedures and outcome compared to anterior circulation stroke in the prospective multicentre REVASK registry. Eur J Neurol.

[28] Alawieh A, Vargas J, Turner RD, Turk AS, Chaudry MI, Lena J, Spiotta A (2018). Equivalent favorable outcomes possible after thrombectomy for posterior circulation large vessel occlusion compared with the anterior circulation: the MUSC experience. J Neurointerv Surg.

[29] Huo X, Raynald Raynald, Gao F (2020). Characteristic and prognosis of acute large vessel occlusion in anterior and posterior circulation after endovascular treatment: the ANGEL registry real world experience. J Thromb Thrombolysis.

[30] Alonso de Leciñana M, Kawiorski MM, Ximénez-Carrillo Á (2017). Mechanical thrombectomy for basilar artery thrombosis: a comparison of outcomes with anterior circulation occlusions. J Neurointerv Surg.

[31] Meinel TR, Kaesmacher J, Chaloulos-Iakovidis P (2019). Mechanical thrombectomy for basilar artery occlusion: efficacy, outcomes, and futile recanalization in comparison with the anterior circulation. J Neurointerv Surg.

[32] Hu SY, Yi HJ, Lee DH, Hong JT, Sung JH, Lee SW (2017). Effectiveness and safety of mechanical thrombectomy with stent retrievers in basilar artery occlusion: comparison with anterior circulation occlusions. J Korean Neurosurg Soc.

[33] Singh RK, Chafale VA, Lalla RS (2017). Acute ischemic stroke treatment using mechanical thrombectomy: a study of 137 patients. Ann Indian Acad Neurol.

[34] Uno J, Kameda K, Otsuji R (2020). Mechanical thrombectomy for basilar artery occlusion compared with anterior circulation stroke. World Neurosurg.

[35] Wang F, Wang J, He Q, Wang L, Cao Y, Zhang H, Xu Z (2020). Mechanical thrombectomy for posterior circulation occlusion: a comparison of outcomes with the anterior circulation occlusion - a meta-analysis. J Atheroscler Thromb.

[36] Zhao W, Ma P, Zhao W (2020). The safety and efficacy of mechanical thrombectomy in posterior vs. Anterior emergent large vessel occlusion: a systematic review and meta-analysis. J Stroke Cerebrovasc Dis.

[37] Adusumilli G, Pederson JM, Hardy N (2022). Mechanical thrombectomy in anterior vs. posterior circulation stroke: a systematic review and meta-analysis. Interv Neuroradiol.

[38] Mbroh J, Poli K, Tünnerhoff J (2021). Comparison of risk factors, safety, and efficacy outcomes of mechanical thrombectomy in posterior vs. anterior circulation large vessel occlusion. Front Neurol.

[39] Mortimer AM, Bradley M, Renowden SA (2012). Endovascular therapy for acute basilar artery occlusion: a review of the literature. J Neurointerv Surg.

[40] Baird TA, Muir KW, Bone I (2004). Basilar artery occlusion. Neurocrit Care.

[41] Zürcher E, Richoz B, Faouzi M, Michel P (2019). Differences in ischemic anterior and posterior circulation strokes: a clinico-radiological and outcome analysis. J Stroke Cerebrovasc Dis.

[42] Argentino C, De Michele M, Fiorelli M (1996). Posterior circulation infarcts simulating anterior circulation stroke. Perspective of the acute phase. Stroke.

[43] Richoz B, Hugli O, Dami F, Carron PN, Faouzi M, Michel P (2015). Acute stroke chameleons in a university hospital: risk factors, circumstances, and outcomes. Neurology.

[44] Wardlaw JM, Murray V, Berge E, del Zoppo GJ (2014). Thrombolysis for acute ischaemic stroke. Cochrane Database Syst Rev.

[45] Merwick Á, Werring D (2014). Posterior circulation ischaemic stroke. BMJ.

[46] Sparaco M, Ciolli L, Zini A (2019). Posterior circulation ischaemic stroke-a review part I: anatomy, aetiology and clinical presentations. Neurol Sci.

[47] Sarikaya H, Arnold M, Engelter ST (2011). Outcomes of intravenous thrombolysis in posterior versus anterior circulation stroke. Stroke.

[48] Lindgren A, Norrving B, Rudling O, Johansson BB (1994). Comparison of clinical and neuroradiological findings in first-ever stroke. A population-based study. Stroke.

[49] Dorňák T, Král M, Hazlinger M (2015). Posterior vs. anterior circulation infarction: demography, outcomes, and frequency of hemorrhage after thrombolysis. Int J Stroke.

[50] Gory B, Eldesouky I, Sivan-Hoffmann R (2016). Outcomes of stent retriever thrombectomy in basilar artery occlusion: an observational study and systematic review. J Neurol Neurosurg Psychiatry.

[51] Szmygin M, Sojka M, Pyra K (2021). Mechanical thrombectomy for acute ischemic stroke in the posterior circulation: assessment of efficacy and outcome and identification of prognostic factors. Acta Radiol.

[52] Demel SL, Broderick JP (2015). Basilar occlusion syndromes: an update. Neurohospitalist.

